# 5-methyl-cytosine and 5-hydroxy-methyl-cytosine in the genome of *Biomphalaria glabrata*, a snail intermediate host of *Schistosoma mansoni*

**DOI:** 10.1186/1756-3305-6-167

**Published:** 2013-06-06

**Authors:** Sara Fneich, Nolwenn Dheilly, Coen Adema, Anne Rognon, Michael Reichelt, Jan Bulla, Christoph Grunau, Céline Cosseau

**Affiliations:** 1 Université de Perpignan Via Domitia, Perpignan, F-66860, France; 2 UMR 5244 CNRS Ecologie et Evolution des Interactions (2EI), Université de Perpignan Via Domitia, 52 Avenue Paul Alduy, Perpignan, Cedex 66860, France; 3 Abt. Biochemie, Max-Planck-Institut für Chemische Ökologie, Jena, D-07743, Germany; 4 Center for Evolutionary and theoretical Immunology, Biology, University of New Mexico, Albuquerque, New Mexico, 87131, USA; 5 LMNO, CNRS UMR 6139, Université de Caen, Caen, Cedex 14032, France

**Keywords:** *Biomphalaria glabrata*, Gastropods, CpG methylation, Epigenetic, *Schistosoma*

## Abstract

**Background:**

*Biomphalaria glabrata* is the mollusc intermediate host for *Schistosoma mansoni*, a digenean flatworm parasite that causes human intestinal schistosomiasis. An estimated 200 million people in 74 countries suffer from schistosomiasis, in terms of morbidity this is the most severe tropical disease after malaria. Epigenetic information informs on the status of gene activity that is heritable, for which changes are reversible and that is not based on the DNA sequence. Epigenetic mechanisms generate variability that provides a source for potentially heritable phenotypic variation and therefore could be involved in the adaptation to environmental constraint. Phenotypic variations are particularly important in host-parasite interactions in which both selective pressure and rate of evolution are high. In this context, epigenetic changes are expected to be major drivers of phenotypic plasticity and co-adaptation between host and parasite. Consequently, with characterization of the genomes of invertebrates that are parasite vectors or intermediate hosts, it is also essential to understand how the epigenetic machinery functions to better decipher the interplay between host and parasite.

**Methods:**

The CpGo/e ratios were used as a proxy to investigate the occurrence of CpG methylation in *B. glabrata* coding regions. The presence of DNA methylation in *B. glabrata* was also confirmed by several experimental approaches: restriction enzymatic digestion with isoschizomers, bisulfite conversion based techniques and LC-MS/MS analysis.

**Results:**

In this work, we report that DNA methylation, which is one of the carriers of epigenetic information, occurs in *B. glabrata*; approximately 2% of cytosine nucleotides are methylated. We describe the methylation machinery of *B. glabrata*. Methylation occurs predominantly at CpG sites, present at high ratios in coding regions of genes associated with housekeeping functions. We also demonstrate by bisulfite treatment that methylation occurs in multiple copies of Nimbus, a transposable element.

**Conclusions:**

This study details DNA methylation for the first time, one of the carriers of epigenetic information in *B. glabrata*. The general characteristics of DNA methylation that we observed in the *B. glabrata* genome conform to what epigenetic studies have reported from other invertebrate species.

## Background

Epigenetic information is information on the status of gene activity that is heritable, for which changes are reversible and that is not based on the DNA sequence. This information can be based on chromatin marking systems such as DNA methylation, histone modifications, and non-coding RNA and the gene location in the nucleus [1-3]. Chromatin exists either as a relaxed structure that is permissive to gene expression and is called euchromatin, or as a condensed structure that is typically silent and is called heterochromatin [1]. Transitions between these different chromatin states may occur in response to environmental signals and result in modification of gene expression, which ultimately, influences phenotypic outcomes without changes in DNA sequence. This environmentally responsive aspect of the chromatin marking system make the Epigenetic Inheritence System a mediator that could allow for rapid adaptive evolution [2,3]. Among the different carriers of the chromatin regulatory system, DNA methylation is one of the best studied. It is conserved in most major eukaryotic groups [4,5], although it has been lost in certain model organisms as listed below [6-8]. The levels, patterns and function of DNA methylation vary among species. In vertebrates and flowering plants, DNA methylation is uniformly distributed in the genome and occurs in transposons (or transposable elements; TE) and repeat elements, in intergenic regions and in transcriptional units including exons and introns. Methylation of promoter sequences has been shown to modulate gene expression, and genetic mobile elements can be silenced through transient methylation [4,5]. Methylation influences important traits such as DNA repair and stability, X chromosome inactivation, inheritable genome imprinting, germ cell pluripotency, as well as development, maintenance and fate of cells.

In invertebrates, the level of DNA methylation varies drastically among species. Some organisms display little or no methylated DNA such as *Tribolium castaneum*, *Drosophila melanogaster*[4] and *Caenorabditis elegans*[8], however, recent investigations have revealed that DNA methylation occurs in many taxa [9-13]. These studies show that genomes of invertebrates are characterized by interspaced regions of methylated and unmethylated DNA. Moreover, intragene (exon and intron) methylation is a general feature of invertebrate organisms [4,5,10,14], while methylation of TE and repetitive elements occur only at moderate levels. Conservation of highly methylated genes is a common feature of invertebrate genome evolution [15], contrasting with plants, for instance, where this is uncommon [16]. Several studies have demonstrated that methylation is likely to occur in genes associated with metabolism and housekeeping functions [11,15], while other studies have suggested that DNA methylation may regulate gene expression by altering the landscape of splice variants [10,17]. In social insects, important traits have been linked to DNA methylation such as those associated with the social roles in the colony [18-20]. In conclusion, DNA methylation is an important feature of the epigenetic control of the genome. At the same time it appears to be relatively heterogeneous in its taxonomical distribution and it can work through different mechanisms to fulfill even different tasks in different taxa. In a first step towards the study of the contribution of epigenetic mechanisms to the interaction of the human parasite *Schistosoma mansoni* with its intermediate gastropod host, we addressed the question of the type of DNA methylation that occurs in the freshwater snail *Biomphalaria glabrata*. *Schistosoma mansoni* is a digenean flatworm parasite that causes human intestinal schistosomiasis. An estimated 200 million people in 74 countries suffer from schistosomiasis and it is the most severe tropical parasitic disease in terms of morbidity after malaria [21]. Eggs of the parasite lodge in a definitive host’s liver (human or rodent) and accumulate to cause serious disease symptoms. With its feces, the vertebrate host releases many schistosome eggs. When these come into contact with water, free-swimming miracidia hatch and actively seek *B. glabrata* snails (and other species depending on geographic location). After penetration into this intermediate host, the parasite develops into sporocysts that produce cercariae [22]. The latter parasite stage is released into water and can infect the vertebrate host. *Biomphalaria glabrata* belongs to one of the largest invertebrate phyla, the Mollusca, which are lophotrochozoans, a lineage of animal evolution distinct from ecdysoans, represented by model invertebrates such as *Caenorhabditis* and *Drosophila*. We describe here that DNA methylation occurs in *B. glabrata*, with approximately 2% of cytosine nucleotides being methylated. Methylation occurs predominantly at CpG sites and it is found in coding regions of genes associated with housekeeping functions. Methylation was also detected in multiple copies of Nimbus, a transposable element.

## Methods

### Ethics statement

The French Ministère de l’Agriculture et de la Pêche and French Ministère de l’Education Nationale de la Recherche et de la Technologie provided permit A 66040 to our laboratory for experiments on animals and certificate for animal experimentation (authorization 007083, decree 87–848 and 2012201-0008)) for the experimenters. Housing, breeding and animal care followed the national ethical requirements.

### Biological material, preparation of genomic DNA

*Biomphalaria glabrata* were reared in spring water at 25°C, 12 hours light / 12 h dark. Genomic DNA was prepared from soft tissues of the foot of pooled snails. The biological samples for methyl cytosine positive and negative controls were obtained from *Oncorhynchus mykiss* (see [23]), Hela cells and from *S. mansoni* adult worms Brazilian strain (*Sm* Bre; see Theron *et al.* 1997). *S. mansoni* indeed displays no or very low levels of DNA methylation [24-26]. HeLa cells were a generous gift of Albertina De Sario INSERM U827 (IURC). Cells were maintained in DMEM-Glutamax (Gibco) with 10% feral calf serum and penicillin/streptomycin.

Tissues or Hela cells were incubated overnight at 55°C with 1 ml of lysis buffer (20 mM Tris/Cl pH 8; 1 mM EDTA; 100 mM NaCl; 0.5% SDS) and 0.3 mg of proteinase K. The samples were then extracted twice with equal volumes of phenol/chloroform, followed by two extractions with equal volumes of chloroform. DNA was precipitated with an equal volume of isopropanol/sodium acetate (3 M, pH5.2) at room temperature. After centrifugation and washing with 1 ml of 70% ethanol, the pellet was dissolved in 100 μl of 1 mM Tris/HCl, pH 8. Finally, DNA was purified using Wizard SV Gel PCR Clean Up System (Promega).

### Expressed sequence data resources

The RNA-seq data from 3 different *B. glabrata* isolates (whole snail): a Guadeloupian strain (*Bg* Gua), two Brazilian strains (*Bg* Bre and Bg Bar) were provided by Guillaume Mitta (personal communication) for use in this study. Part of the analysis was performed with the RNA-seq data publicly available from snaildb (http://www.snaildb.org/content/blast) [27].

### Correlation tests

For correlation analysis between the level of transcript expression and the quantity of CpG methylation of these respective transcripts, we performed a correlation test between the transcript rpkm results (Guillaume Mitta, Personal communication) and their respective CpGo/e ratio. Both a linear (Pearson’s) and non-linear (Spearman’s) correlation analysis were performed using the R 2.15.2 (2012 The R Foundation for Statistical Computing ISBN 3-900051-07-0) under a local Galaxy instance [28].

### Blast search for molecular machinery for DNA methylation

To investigate the presence of the genes for the molecular machinery for DNA methylation in *B. glabrata*, we performed tblastN against the supercontigs of the preliminary genome assembly version 4.3 (http://www.biology.unm.edu/biomphalaria-genome/index.html). We used as a query, sequences from *Homo sapiens* for which the annotation corresponded to different categories of relevant protein families: DNMTs (DNA Methyl Transferase), Tet (Ten Eleven Translocation enzymes) and MBDs (Methyl-CpG Binding Domain proteins). Expression of each candidate was tested in the different transcript databases (http://www.snaildb.org/content/blast) and the 3 RNA-seq libraries generated in our lab from different isolates of *B. glabrata*.

*B. glabrata* potential MBD2/3 protein sequence was aligned with the BioEdit software (http://www.mbio.ncsu.edu/bioedit/bioedit.html), with the following proteins sequences: *Apis mellifera* MBD2/3 (XP_392422.2), *Homo sapiens* MBD2 (NP_003918.1), *H. sapiens* MBD3 (NP_003917.1), *Hemicentrotus pulcherrimus* MBD2/3 (ACF05485.1), *Crassostrea gigas* MBD2/3 (EKC32831.1), *Ixodes scapularis* MBD2/3 (XP_002407962.1).

### Digestion with methylation sensitive restriction enzymes

After DNA extraction, restriction digests with *Hpa*II (R0171S, 10000 units/ml, Bio*Labs*) and *Msp*I (R0106S, 20000 units/ml, Bio*Labs*) were carried out for two hours at 37°C with 200 ng of genomic DNA and 6 units of enzyme in a final volume of 20 μl. *Msp*I and *Hpa*II share the same CCGG recognition site but the latter enzyme cannot cut sites with methylated cytosines. DNA fragments were size separated by electrophoresis through a 1% agarose/TBE gel and visualised using ethidium bromide staining.

### Liquid chromatography- mass spectrometry (LC-MS) analysis

The levels of 5-methylcytosine (5-mC) and 5-hydroxymethylcytosine (5-hmC) in DNA samples were determined by LC-MS/MS analysis as their deoxyribonucleosides. Pooled genomic DNA of 10 individuals was digested to nucleosides with DNA Degradase Plus (E2020, Zymo research). Fifty units of the enzyme preparation were added to 10 μg of DNA in a total volume of 100 μl. Samples were incubated at 37°C for one hour with low speed rotation. An additional 50 units of enzyme were added to each sample and digestion was continued for one more hour. Complete digestion into nucleosides was verified by visualization of the digest on a 1% agarose gel. Nucleic acid concentration was quantified spectrophotometrically (Nanodrop, Thermo Scientific). Finally, samples were lyophilised and resuspended in 200 μl of water. A 2 μl aliquot was injected into an LC-MS/MS system for analysis of 5-methyl-2′-deoxycytidine and 5-hydroxymethyl-2′-deoxycytidine, and a 2 μl aliquot of a 1:50 (v:v) diluted sample (dilution in water) was processed for analysis of 2′-deoxycytidine. Chromatography was performed on an Agilent 1200 HPLC system (Agilent Technologies, Boeblingen, Germany) with a Zorbax Eclipse XDB-C18 column (50 × 4.6 mm, 1.8 μm, Agilent Technologies, Germany). Formic acid (0.05%) in water and acetonitrile were employed as mobile phases A and B respectively. The elution profile was: 0-0.5 min, 5% B; 0.5-2 min, 5-100% B in A; 2-3 min 100% B in A; 3-3.1 min 100-5% B and 3.1-5.5 min 5% B. The mobile phase flow rate was 1.1 ml/min, column temperature was maintained at 25°C. The HPLC was coupled to an API 5000 tandem mass spectrometer (Applied Biosystems, Darmstadt, Germany) equipped with a Turbospray ion source operated in positive ionization mode. The instrument parameters were optimized by infusion experiments with pure standards: 2′-deoxycytidine (Acros Organics, New Jersey, USA); 5-methyl-2′-deoxycytidine (ABCR, Karlsruhe, Germany); 5-hydroxymethyl-2′-deoxycytidine (Berry & Associates, Ann Arbor, USA). The ionspray voltage was maintained at 5500 eV. The turbo gas temperature was set at 700°C. Nebulizing gas was set at 70 psi, curtain gas at 25 psi, heating gas at 60 psi and collision gas at 6 psi. Multiple reaction monitoring (MRM) was used to monitor analyte parent ion → product ion: *m/z* 228 → 112 (collision energy (CE) 17 V; declustering potential (DP) 45 V) for 2′-deoxycytidine; *m/z* 242 → 126 (CE 15 V; DP 51 V) for 5-methyl-2′-deoxycytidine; *m/z* 258 → 142 (CE 15 V; DP 41 V) for 5-hydroxymethyl-2′-deoxycytidine. Both Q1 and Q3 quadrupoles were maintained at unit resolution. Analyst 1.5 software (Applied Biosystems, Darmstadt, Germany) was used for data acquisition and processing. Linearity in ionization efficiencies were verified by analyzing dilution series of authentic standards. External calibration curves for 2′-deoxycytidine, 5-methyl-2′-deoxycytidine, and 5-hydroxymethyl-2′-deoxycytidine were used for quantification.

### Bisulfite treatment, PCR amplification, COBRA and sequencing

Bisulphite sequencing allows identifying DNA methylation at single nucleotide resolution. The treatment of DNA with bisulfite converts all non-methylated cytosines into deoxy-uracil while the 5-methylcytosines (5-mC) and 5-hydroxymethyl-cytosines (5-hmC) remain intact. To address the question whether repetitive elements are methylated and to confirm the occurrence of CpG methylation in transcripts in *B. glabrata*, we analysed bisulfite treated gDNA by PCR targeting the non-LTR retrotransposon nimbus (*BgI*). *BgI* is transcriptionally active in different stages of the mollusc development, and at least 100 copies were detected in the *B. glabrata* genome [29,30]. Bisulfite treatment was carried out as described previously [31,32] (http://www.methdb.univ-perp.fr/epievo/). Briefly, 300 ng DNA was denaturated with 3 M NaOH, treated with a solution of sodium-bisulfite and hydroquinone at pH 5 in the dark for 4 hours at 55°C; desalted (Amicon Ultra column, UFC510024 Millipore), desulfonated by adding 350 μl of 0.1 M NaOH to the DNA in the Microcon column, and finally dissolved in 10 mM Tris/Cl pH 8. The genomic sequence of the transposable element Nimbus *BgI* was obtained from Genbank (acc.nr. EF413180, [29]). Primer pairs (Table 1) were designed using MethPrimer [33]. Six ng of treated DNA were used as a template for PCR amplification in 25 μl using Go Taq DNA polymerase (Promega) at 94°C for 2 min, 5 cycles of 94°C for 1 min, 50°C for 2 min and 72°C for 3 min; followed by 25 cycles of 94°C for 30 sec, 50°C for 2 min and 72°C for 1:30 min and finally 72°C for 10 min. PCR products were separated by electrophoresis through 1% agarose gels. The degree of methylation was estimated by COmbined Bisulfite Restriction Assay (COBRA) [34]. Suitable restriction enzymes were selected with Snake Charmer (http://insilico.ehu.es/restriction/two_seq/snake_charmer.html) and used to digest each of the PCR products by three different enzymes: *Aci*I (NEB); *Tai*I (Fermentas); *Taq*I (Promega) using 15 units to digest 100 ng in each reaction. Digestion fragments were separated on a 2% agarose gel. For high resolution analysis, 1 μl of each PCR product was cloned into pCR4 (TOPO TA Cloning kit, Invitrogen) and 11 inserts derived from *Bg* Bre and *Bg* Gua DNA each were sequenced with vector specific primers (M13F: CTGGCCGTCGTTTTAC and M13R: CAGGAAACAGCTATGAC) using the GATC Biotech facilities (http://www.gatc-biotech.com/fr/entreprise/gatc.html). Chromatograms were edited using Sequencher Software, and a total of 14 sequences (combined from *Bg* Bre and *Bg* Gua) were retained for final analysis. These experimentally obtained sequences were aligned with the genomic sequence from GenBank (Bioedit) to visualise the sites of methylated cytosine. MethTools 2.0 software [35] was used to generate a graphical view of the *BgI* region 2 containing the methylated sites.

**Table 1:** Primers used in this study

**Region**	**Primer names**	Sequence and 5′ position*		**Amplicon length**
		**Forward**	**Reverse**	
Region 1	Nimbus 1	5′-TTTTATGAGGTGTTTTAAGTGTTAGG-3′ (5′ position: 1132)	5′-AAAAATTTCCCTTTATTCCAATAAC-3′ (5′ position: 1917)	785 bp
Region 2	Nimbus 2	5′-TTGGATGTTAAAATTTTTGTTAGAA-3′ (5′ position: 2986)	5′-AAAAAATATCCCTTAAACCCCATAA-3′ (5′ position: 3871)	785 bp
Region 3	Nimbus 3	5′-ATTTTAGGGAATTGTAGGAGAGTTA-3′ (5′ position: 4802)	5′-CTTATCAAACCCTTAAATATAAACC-3′ (5′ position: 5373)	571 bp

*position is given on the genbank sequence: EF413180.

### CpG observed/expected ratio analysis

The CpG observed/expected ratio (CpGo/e) analysis was performed on cDNA sequences (≥500 bp) of 4 different RNA-Seq assemblies: 1 public dataset (http://www.snaildb.org/webfm), representing 12579 uniseq from the *Bg* Lilles strain [27] and 4 libraries generated in our laboratory from different geographical isolates of *B. glabrata* (42824 uniseq - strain: *Bg* Bre, 22088 uniseq – strain: *Bg* Gua and 16855 uniseq – strain: *Bg* Bar). The CpGo/e ratio was used as a proxy to estimate the intragene DNA methylation content of *B. glabrata* as previously described in other invertebrate species [11,36,37]. CpGo/e was calculated from 5000 uniseq randomly chosen in each RNA-seq library. CpGo/e was calculated for the coding strand only, using the following equation where l is the number of nucleotides [11].

$$\mathrm{CpGo}/e=\frac{\mathrm{number}.\mathrm{of}.\mathrm{CpG}}{\mathrm{number}.\mathrm{of}.C\times \mathrm{number}.\mathrm{of}.G}\times \frac{l^{2}}{l-1}$$

To discern two subgroups with high and low CpGo/e values, statistical analyses were carried out using the software R 2.15.2 (http://www.r-project.org). The clustering algorithm employed utilizes the Mclust procedure from the package mclust, version 4.0 [38,39]. Akaike information criterion (AIC) and Bayesian information criterion (BIC) were used as selection criteria to establish if a simple Gaussian or a mixture of two Gaussian distributions fit best to empirical distribution of CpGo/e values of *B. glabrata* (Gua strain). The 95% confidence intervals for the mean values were determined by non-parametric bootstrap with 1000 replications.

### Functional analysis on low versus high methylated genes

Transcripts from the *Bg* Gua transcriptomic library were chosen for further functional analysis. Functional annotation was performed on the *Bg* Gua data set (22088 unisequences > =500 pb) using Blast2GO version 2.4.2 [40]. The pipeline annotation and parameters used were as follows: i) an initial annotation with BLASTX (against the nonredundant NCBI database; e-value at 1.10^-6^); ii) a protein domain search using InterProscan; iii) an enzyme annotation using the Kyoto Encyclopedia of Genes and Genomes (KEGG); and iv) assignment of a Gene Ontology term (GO; http://www.geneontology.org/).

*Bg* Gua cDNA sequences (> = 500 pb) were divided into 11 cDNA subsets based on their CpGo/e ratio (0-0.1: 1283 uniseq, 0.1-0.2: 3878 uniseq, 0.2-0.3: 4345 uniseq, 0.3-0.4: 3106 uniseq, 0.4-0.5: 2165 uniseq, 0.5-0.6: 1669 uniseq, 0.6-0.7: 1665 uniseq, 0.7-0.8: 1425 uniseq, 0.8-0.9: 989 uniseq, 0.9-1: 788 uniseq, >1: 775 uniseq). The cDNA set from each subcategory was independently tested (exact Fisher test, p-value < 0.05, two-tailed) for enrichment in functional categories (GO terms) against the complete *Bg* Gua transcriptome set (> = 500 pb, 22088 uniseqs). Over-represented or under-represented GO terms were classified in 3 subcategories independently by 5 experienced biologists: housekeeping GO, non informative GO, inductible GO. GO terms indicating a localization (for example: nucleolus) or a general function (for example: ATP binding) that could either be involved in housekeeping functions or inducible functions were considered as non informative.

## Results

### Essential elements of the DNA methylation machinery are present in B. glabrata

The DNA methyltransferase (DNMT) proteins are a family of enzymes that catalyse the transfer of a methyl group to the carbon atom number 5 of the cytosin moiety. Three classes of DNMTs have been characterized in mammalians. DNMT1 is responsible for the maintenance of methylation and preferentially methylates hemimethylated CpG di-nucleotides. DNMT2 has strong sequence similarities with 5-methylcytosine methyltransferases but has only been shown to methylate tRNA and not DNA [41]. Therefore, its role as a DNA methyl transferase per se remains enigmatic. DNMT3 is responsible for *de novo* CpG methylation and occurs in all mammalian and some invertebrate species. We investigated the occurrence of these 3 enzymes in *B. glabrata* by BLAST search against the preliminary assembly v4.3 of the *B. glabrata* genome. The supercontigs 19819.1, 15589.2 and 15589.1 contain protein-encoding sequences similar to the carboxy terminal, central and amino terminal part of the human DNMT1 sequence, respectively, suggesting that one gene encoding DNMT1 is present in the *B. glabrata* genome (Additional file 1). The supercontig 6274.3 contains genomic sequences encoding a protein that is conserved with the DNMT2 query sequence. Partial transcripts corresponding to both the DNMT1 and DNMT2 candidates were detected in the *B. glabrata* RNAseq libraries. Therefore, the *B. glabrata* genome includes unique genes encoding DNMT1 and DNMT2 homologs, and these are expressed as transcripts. No sequence matches the DNMT3 query sequence, either in the genome draft or in the RNA seq libraries.

The Ten Eleven Translocation enzymes (Tet1, 2, 3) are a family of oxygenases that catalyse the conversion of 5-methylcytosine (5-mC) into 5-hydroxymethylcytosine 5-hmC in DNA. 5-hmC has long been believed to be an intermediate in the DNA demethylation pathway but recent evidence indicates that its role in epigenetics may have been underrated so far [42,43]. We performed tBlastN against the preliminary genome assembly and found that two *B. glabrata* supercontigs (Contig18827.1 and Contig21.52) contain sequences that match the oxygenase domains present in tet enzymes but that lack the amino terminal part of the tet human orthologs (Additional file 1). The % of coverage of the query sequence is only 50%. No transcripts with similarity to tet enzymes were detected in the RNA-seq libraries. Therefore, it remains an open question whether true “tet enzymes” or unrelated proteins with oxygenase activity occur in *B. glabrata*.

The MBD2 and MBD3 (Methyl-CpG Binding Domain proteins) query sequences match with the same unique target in the *B. glabrata* genome, which is located on contig 7228 (Additional file 1). This would be consistent with studies that report that other invertebrate species differ from vertebrates in that they possess only one gene that combines the function of MBD2 and MBD3 proteins, whereas homologs of MBD1 and 4 are not detected [44]. All four RNA-seq libraries included full length transcripts of this sequence, therefore the MBD2/3 gene could be reconstituted and sequence alignments indicate closed conservation with MBD2/3 sequences from other invertebrate species (Additional file 2).

The available data indicate that critical elements of the machinery for DNA methylation are present, suggesting that *B. glabrata* DNA could be methylated.

### Genomic DNA of B. glabrata is only partially digested with methylation sensitive restriction enzymes

**Figure 1:**
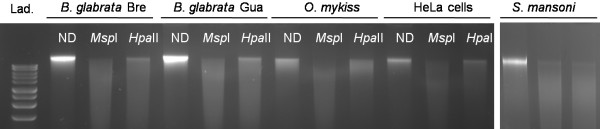
**Methylation sensitive restriction assay.** Genomic DNA of *B. glabrata:* Brazilian strain (*B. glabrata Bre*) and Guadeloupian strain (*B. glabrata Gua*), of positive controls: *O. mykiss and* HeLa cells and of negative controls: *S. mansoni* (miracidia) was digested with the *Msp*I enzyme, its methylation sensitive isoschizomer *Hpa*II or was not digested (ND). 200 ng of DNA were loaded per well. Digestion profile was observed on an ethidium bromide stained 1% agarose gel. Lad: 1 kb DNA ladder (promega), lowest band 1500 bp, highest band 10000 bp.

Digestion of *B. glabrata* gDNA with methylation sensitive *Hpa*II (restriction site 5′-CCGG-3′) revealed an undigested high molecular weight fraction as compared to the continuous smear of digestion products produced by its isoschizomer *Msp*II that is insensitive to methylation (Figure 1). In contrast, gDNA from *S. mansoni* that contains very low levels of methyl cytosine [24-26], was digested to completion with both *Msp*II or *Hpa*II: no undigested high molecular weight fraction remained. The DNA from *O. mykiss* and Hela cells (positive controls) displayed a more pronounced resistance to *Hpa*II digestion, consistent with the relatively high levels of CpG methylation observed in these organisms [23]. The resistance to *Hpa*II digestion provides an indication for methylation of CpG in restriction sites present in the genomic DNA of *B. glabrata*. The gel aspects of the restriction digests were similar for two different strains of *B. glabrata* species; indicating no obvious differences in CpG methylation profiles for these two strains.

### Two percent of total cytosines are methylated in B. glabrata

For a precise measurement of the amount of methylated and hydroxymethylated cytosines, genomic DNA of *Bg* Gua and *Bg* Bre was digested into nucleosides and the occurrence of mC and hmC was analysed by LC-MS/MS. The results show that in the *B. glabrata* genome, about 2% of total cytosines are methylated and 0.0009% of total cytosines are hydroxymethylated (Table 2). The control, consisting of an unmethylated PCR product tested negative for either type of modified cytosines.

**Table 2:** **5-methylcytosine (5-mC) and 5-hydroxymethylcytosine (5-hmC) levels in *B. glabrata *strains as determined by LC/MS**

***B. glabrata *strains**	**% 5-mC**	**% 5-hmC**
Guadeloupian strain	2.1	0.00091
Brazilian strain	2.07	0.0009

### B. glabrata transcripts are divided in low and high methylated genes

**Figure 2:**
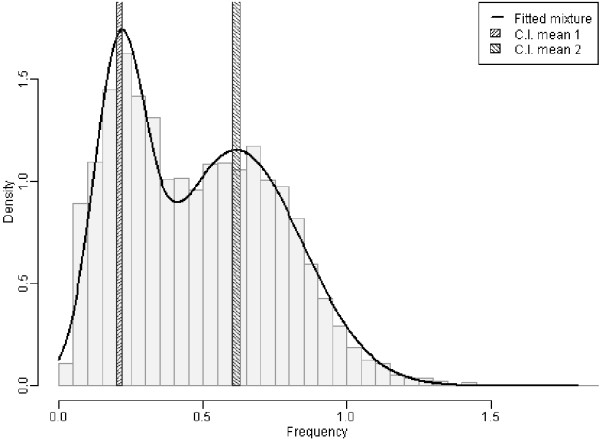
**Histogram of CpGo/e ratio in *B. glabrata *transcripts.** CpGo/e ratio was measured as a proxy to estimate the CpG methylation in transcripts from RNA-seq libraries from *B. glabrata* guadeloupian strain (*Bg* Gua). X axis: CpGo/e ratio, Y-axis density of transcripts. The figure displays a histogram of *Bg* Gua CpGo/e ratios with a fitted mixture distribution. The grey shaded bars represent 95% confidence intervals for the two mean values. The estimated mean values of the two components are 0.209 and 0.616.

We calculated the empirical distribution of CpGo/e ratios for the transcripts from the 3 RNAseq libraries issued from different geographical areas and from the recently published library from the strain *Bg* Lille [27]. The profile from all the libraries was the same (data not shown). A mixture of two Gaussian distributions as well as a simple Gaussian distribution were fitted to the CpGo/e ratio data from the *Bg* Gua library. The model selection criteria AIC and BIC both indicate a clear preference for the mixture model. The AIC values equal 1243 and 386 for the simple Gaussian and the mixture model respectively and the BIC values equal 1256 and 419 for the simple Gaussian and the mixture model respectively. Figure 2 displays a histogram of *Bg* Gua CpGo/e ratios with the fitted mixture distribution. The estimated mean values of the two components are 0.209 and 0.616. Their 95% confidence intervals equal [0.201, 0.218] and [0.604, 0.628], respectively. The two clearly distinct means with non-overlapping confidence intervals underline the bi-modality, indicating the presence of two sub-populations in the sample: presumably high-methylated genes and presumably low methylated-genes. The proportions of these two potential sub-populations can be inferred a posteriori as by-products of the parameter estimation procedure: the corresponding values equal 33.3% and 66.7% for the component with lower and higher means, respectively.

### Gene methylation categories are correlated with functions of the gene products but not with transcription level

We retrieved the lists of transcripts that display CpGo/e ratios between 0-0.1, 0.1-0.2, 0.2-0.3, 0.3-0.4, 0.4-0.5, 0.5-0.6, 0.6-0.7, 0.7-0.8, 0.8-0.9, 0.9-1 and >1. Each of these 11 subcategories was submitted to a Fisher exact test in the blast2Go web-based interfaced to test for enrichment in functional categories in each of these subclasses. GO categories (Gene Ontology categories), that were statistically enriched were then classified as housekeeping, non informative or inducible (see Additional file 3 for these assignments). The GO categories over-represented in the subset of CpGo/e ratios from 0.1 to 0.4 were more enriched in housekeeping functions, whereas subsets of CpGo/e ratios from 0.4 to >1 were more enriched in inducible categories (Table 3). We observed a transition for categories in the subset of CpGo/e ratio from 0.4 to 0.6. Expectedly, the GO categories under-represented in the subset of CpGo/e ratio from 0.4 to >1 were more enriched in housekeeping categories (Table 3). Therefore, the high methylated gene category is more enriched in genes encoding housekeeping functions as previously shown for other invertebrates [11].

**Table 3:** **Functional analysis on transcripts depending on their** CpGo/e **ratio**

**CpGo/e ratio**	**Over-represented GO terms**			**Under-represented GO terms**		
	**Housekeeping functions**	**Inducible functions**	**Non informative GO terms**	**Housekeeping functions**	**Inducible functions**	**Non informative GO terms**
0-0.1	No enriched categories			No enriched categories		
0.1-0.2	5	2	5	4	2	3
0.2-0.3	14	1	9	1	3	2
0.3-0.4	12	0	8	0	1	1
0.4-0.5	No enriched categories			No enriched categories		
0.5-0.6	3	1	3	2	1	5
0.6-0.7	0	3	2	No enriched categories		
0.7-0.8	1	8	2	14	1	13
0.8-0.9	0	0	2	7	1	6
0.9-1	0	1	3	2	0	3
>1	7	12	4	1	0	12

*Bg* Gua *cDNA* (> = 500 pb) were divided into 11 *cDNA* subsets based on their *CpGo*/e ratio (Left column: 0-0.1 to >1). The *cDNA* set from each subcategory was independently tested for enrichment in functional categories (*GO* terms) against the complete *Bg* Gua transcriptome set. Over-represented or under-represented *GO* terms were classified in 3 subcategories: housekeeping *GO*, non informative *GO*, inductible *GO* (See Additional file 3 for assignment in these subcategories). The numbers represent the total number of *GO* terms that belong to one of these subcategories.

We tested whether *Biomphalaria* DNA methylation is related to transcript expression. CpGo/e ratio versus rpkm correlation analysis did not give a significant p-value for both Pearson’s and Spearman’s correlation analysis. Therefore, we did not find a significant genome-wide correlation between transcript CpG methylation and gene expression.

### Many copies of the B. glabrata non-LTR retrotransposon nimbus (BgI) are highly methylated

**Figure 3:**
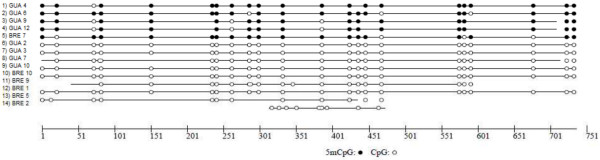
**CpG methylation site in the non-LTR retrotransposon nimbus (*BgI*).** Schematic representation of CpG methylation in 22 CpG sites of 14 DNA molecules of the 751 bp fragment of *BgI* in *B. glabrata* (*Bg* Bre and *Bg* Gua). Data was investigated by bisulfite sequencing. Black and white circles correspond to methylated and non-methylated CpGs, respectively. No methylation was detected outside CpGs.

To further analyze whether DNA methylation also occurs in intergenic regions and in particular in repetitive sequences as represented by a retrotransposon like Bg1 Nimbus, we used bisulfite conversion based techniques. After bisulfite treatment of genomic DNA from *B. glabrata*, three different regions of the transcriptionally active non-LTR retrotransposon nimbus (*BgI*) were amplified by PCR from each *B. glabrata* strain. Then, digestion of this PCR product with restriction enzymes that target sites containing cytosine (*Aci*I, *Tai*I or *Taq*I), indicated methylation in *BgI* region 2. It indicates that this region has methylated cytosines. For high resolution mapping of the methylated sites, 14 amplicon sequences of region 2 (mixed *Bg* Gua and *Bg* Bre) were cloned and sequenced. Alignments of these sequences allowed us to detect that 5 out of the 14 sequences contained methylated CpG sites (Figure 3). A total of 22 methylated CpG sites were identified among the 5 clones (Figure 3).

## Discussion

DNA methylation is a covalent modification that consists of adding a methyl moiety from a folate donor to the carbon atom number 5 of cytosine. This modification is an important epigenetic determinant involved in control of gene expression, maintenance of DNA integrity and stability, one that can be inherited through cell division. While the role of this epigenetic modification has been intensively studied in vertebrate species, plants and invertebrates from the ecdysoan lineage [36]*,* literature reports on molecular epigenetic studies in lophotrochozoan protostome invertebrates such as molluscs are rare [11], especially because of the lack of genomic molecular resources for this phyla. Our study provides the first evidence of DNA methylation in the genome of *B. glabrata*, a gastropod mollusc and further expands insights of DNA methylation in the mollusc phylum, previously only reported for the class of bivalve molluscs: oyster *Crassostrea gigas*[11] and the scallop *Chlamys farreri*[45]. We estimate the percentage of methylated cytosines in the genome of *B. glabrata* to be roughly 2% by LC-MS analysis of nucleosides. This rate of DNA methylation is rather high for an invertebrate whose general percentage of cytosine being methylated ranks from 0.1 to 4% [46].

We investigated the presence of molecular components that make up the DNA methylation machinery in *B. glabrata*. DNMT1, involved in maintenance of CpG methylation, and DNMT2, characterised as a tRNA methyl transferase, are present in *B. glabrata,* while DNMT3, involved in *de novo* methylation, seems to be lacking in this organism. This result provides another example of organisms that display a functional methylation system in a genome where DNMT3 is lacking. *Schistocerca gregaria*, *Locusta migratoria*, *Bombyx mori* and *Pediculis humanus* are other invertebrate species for which no DNMT3 has been detected, although they display functional DNA methylation machinery [36,37,47,48]. It should be noted that the absence of DNMT3 is not a general feature in molluscs as DNMT3 is present in *C. Gigas*[11]. For the organisms lacking DNMT3, the question remains open as to what the machinery is for the *de novo* methylation. DNMT1 is a potential candidate; it has been reported to function in the role of a *de novo* methyltransferase in human cancer cells [49]. Other candidates involved in the DNA methylation machinery of *B. glabrata* belong to the family of Methyl-CpG Binding Domain proteins (MBDs). MBDs specifically bind methyl-CpG and interact with the chromatin remodeling complex, which results in other local epigenetic modifications. Four classes of MBDs have been described (MBD1 to 4) [44]. Consistent with observations from other invertebrate species, a single gene encoding for a protein that may combine the function of MBD2 and MBD3 proteins occurs in *B. glabrata* and MBD1 and 4 are not detected [44].

Different types of DNA methylation have been found in invertebrates. Some species dominantly display methylation in the context of CpG, such as *C. intestinalis*, *S. gregaria*, *B. mori*, *M. extradentata* and *A. mellifera*[5,9,14,47,48,50], while other species such as *P. lilacinus* and *D. melanogaster, Pogonomyrmex barbatus* display a high proportion of methylation in non-CpG methylation sites [10,13,51]. The bisulfite sequencing analysis performed in our study identified only methylation of CpG sites, suggesting that this is the dominant type of DNA methylation in *B. glabrata*. The bisulfite sequencing analysis targeted the non LTR-retrotransposon nimbus (*BgI*) [29], a Mobile Genetic Element (MGE) that is constitutively expressed in naïve *B. glabrata* and its transcription is enhanced upon stress conditions (heat shock and *S. mansoni* infection) [30]. An estimated 100 copies of *BgI* are present in the *B. glabrata* genome [29]. We detected 22 sites of CpG methylation in a 752 bp region of *BgI*, and observed variation in the methylation status of the particular CpG sites among the clones that were analysed. Variability of the sources of the *BgI* sequences, different types of cells, developmental stages, individuals and genomic location of *BgI* copies, may be responsible for this variation in methylation of specific CpG sites. Patterns of MGEs methylation vary considerably in invertebrates. In some species, MGEs methylation is modest and not a preferred target (*C. intestinalis*, *Drosophila*, *C. farreri* , *A meliflera* and *B. mori*[4,5,14,48,50,52]), while in other invertebrates repetitive DNA seems to be preferentially methylated (*L. migratoria*, *Medauroidea extradentata* and *S. gregaria*[9,37,47]). These discrepancies question the idea of a conserved role in invertebrates for methylation in suppressing transcription of transposons as has been demonstrated in mammals and flowering plants [46].

Methylated cytosines are hypermutable because they are spontaneously transformed into thymine by deamination [53,54]. This spontaneous mutation is not recognised by the DNA repair machinery and leaves an evolutionary signature in the genome of organisms that display CpG methylation or have displayed CpG methylation in the past and can be identified through *in silico* analysis. Consistently, methylated regions of DNA have a globally reduced frequency of CpG dinucleotides compared with the expected frequency. Consequently, there is a negative correlation between the CpGo/e ratio and the degree of methylation, as has been validated in several insect species [15]. In *B. glabrata*, we evaluated the distribution of CpGo/e ratios among expressed sequences from RNA-seq libraries. We clearly observed that most of the sequences display a CpGo/e ratio below 1, consistent with CpG methylation in the units of transcription (transcribed genes). The presence of intragenic methylation in *B. glabrata* is in line with observations reported from other invertebrate species [4,5,14,15]. Furthermore, we observed a bimodal distribution of the normalised CpG ratio, with two peaks, at 0.21 and 0.69 respectively. This bimodal distribution was also observed previously in several other invertebrate species [15,36,55] and indicates the presence of two categories of genes: Low CpGo/e transcripts corresponding to high-methylated genes and high- CpGo/e transcripts corresponding to low methylated-genes. Functional annotation of these genes shows that transcripts predicted to be highly methylated are associated with housekeeping functions and genes predicted to be poorly methylated are associated with inducible functions. This correlation has already been reported in other invertebrate species such as *C. gigas*[11] and the *A. mellifera*[56].

Epigenetic mechanisms shape the expression of the genome during development. In addition, they provide a source for potentially heritable phenotypic variation and could, therefore, be involved in the adaptation to environmental constraints such as parasitic infections. Consequently, it is indispensible to understand the genome of parasite vectors and intermediate hosts but also how the epigenetic machinery functions. This study provides the first report of a precise estimate for the amount of methylated DNA, one of the carriers of epigenetic information, in *B. glabrata* and we deliver insights into its distribution along the genome. We hope to pave the way for a more thorough analysis of the role of DNA methylation in the susceptibility towards infection by parasites such as *S. mansoni*.

## Abbreviations

LC-MS: Liquid chromatography-mass spectrometry; HPLC: High performance liquid chromatography; MRM: Multiple reaction monitoring; CE: Collision energy; 5-mC: 5-methylcytosine; 5-hmC: 5-hydroxymethylcytosine; MBD: Methyl-CpG binding domain protein; Tet: Ten eleven translocation enzymes; DNMT: DNA methyltransferase; GO: Gene ontology; TBE: Tris-borate-EDTA; CpGo/e: CpG observed/expected ratio; BgI: Non-LTR retrotransposon nimbus; LTR: Long terminal repeats; MGE: Mobile genetic element; AIC: Akaike information criterion; BIC: Bayesian information criterion

## Competing interests

The authors declare that they have no competing interests.

## Authors’ contributions

SF performed the experiments, analysed the data and contributed to preparation of the reagents, the materials and the analysis tools. She also took part in the writing of the manuscript. ND contributed to preparation of RNA-seq libraries and participated in the data analysis. CA took part in the writing of the manuscript. AR contributed to the preparation of the reagents and the biological samples. MR performed the LC-MS experiments. JB designed, performed and described the statistical test that was used to test for bimodal distribution of observed/expected CpG ratios. CG conceived and designed the experiments, analysed the data, contributed to preparation of the analysis tools and wrote the manuscript. CC conceived and designed the experiments, performed the experiments, analysed the data, and wrote the manuscript. All authors read and approved the final version of the manuscript.

## Supplementary Material

Additional file 1 Blast analysis on methylation machinery candidates. Click here for file

Additional file 2 **Sequence alignments of MBD2/3 protein families.***B. glabrata* MBD2/3 protein sequence was aligned with the BioEdit software (http://www.mbio.ncsu.edu/bioedit/bioedit.html) with the following protein sequences: *Apis mellifera* MBD2/3 (XP_392422.2), *Homo sapiens* MBD2 (NP_003918.1), *H. sapiens* MBD3 (NP_003917.1), *Hemicentrotus pulcherrimus* MBD2/3 (ACF05485.1), *Crassostrea gigas* MBD2/3 (EKC32831.1), *Ixodes scapularis* MBD2/3 (XP_002407962.1). Click here for file

Additional file 3 **Classification of GO functional categories in housekeeping, inducible or non informative subcategories.***Bg* Gua cDNA (> = 500 pb) were divided into 11 cDNA subsets based on their CpGo/e ratio. Each subset was tested for enrichment in functional category using the Blast2GO software. Over-represented or under-represented GO terms (p value < 0.05) were classified into 3 subcategories: housekeeping GO (underlined in red), non informative GO (not underlined), inducible GO (underlined in yellow). Click here for file
