# Supplementary material for: 5-methyl-cytosine and 5-hydroxy-methyl-cytosine in the genome of Biomphalaria glabrata, a snail intermediate host of Schistosoma mansoni
Source: Parasit Vectors. 2013 Jun 6;6:167. doi: 10.1186/1756-3305-6-167 (PMC3681652; doi:10.1186/1756-3305-6-167)
Supplement: Additional file 1 — Blast analysis on methylation machinery candidates. [file 1756-3305-6-167-S1.docx]

**Aditionnal file 1: Blast analysis on methylation machinery candidates**

DNMT1 tblastN results (query sequence: AAI26228, 1632AA):

| Localisation on query sequence | Localisation on bg contig sequence | % positives | Conserved domains |
| --- | --- | --- | --- |
| 0-362 |  | <=40% | pfam06464, DMAP1-binding Domain |
| 362-426 | Contig19819.1  2302-2018 | 42% |  |
| 423-467 | Contig19819.1  1666-1532 | 73% | pfam12047 Cytosine specific DNA methyltransferase replication foci domain |
| 467-550 | Contig15589.2  4796-4545 | 80% |  |
| 531-676 | Contig15589.2  4227-3727 | 51% |  |
| 669-706 | Contig15589.2  3189-3076 | 86% | pfam02008, CXXC zinc finger domain |
| 706-769 | Contig15589.2  2082-1876 | 49% |  |
| 776-850 | Contig15589.2  881-660 | 69% | cd04760, Bromo domain, present in DNA (Cytosine-5)-methyltransferases |
| 849-921 | Contig15589.1  26165-25947 | 59% |  |
| 929-978 | Contig15589.1  25431-25279 | 60% | cd04711, Bromo domain, present in DNA (Cytosine-5)-methyltransferases |
| 980-1039 | Contig15589.1  24988-24812 | 76% |  |
| 1039-1120 | Contig15589.1  24549-24304 | 69% |  |
| 1152-1235 | Contig15589.1  23501-23252 | 72% | cl16911, S-adenosylmethionine-dependent methyltransferases |
| 1231-1316 | Contig15589.1  22740-22483 | 96% |  |
| 1309-1370 | Contig15589.1  21975-21787 | 87% |  |
| 1366-1406 | Contig15589.1  21344-21222 | 80% |  |
| 1405-1471 | Contig15589.1  20414-20214 | 88% |  |
| 1473-1552 | Contig15589.1  20118-19882 | 87% |  |
| 1553-1592 | Contig15589.1  19659-19540 | 97% |  |
| 1591-1613 | Contig15589.1  19309-19241 | 60% |  |

DNMT2 tblastN results (query sequence: CAG29312, 391AA):

| Localizations on query sequence | Localisation on bg contig sequence | % positives | Conserved domains |
| --- | --- | --- | --- |
| 4-47 | Contig6274.3  9738-9610 | 68% | cd00315, Cytosine-C5 specific DNA methylases  cl16911S-adenosylmethionine-dependent methyltransferases (SAM or AdoMet-MTase) |
| 22-60 | Contig6274.3  7385-7269 | 69% |  |
| 49-106 | Contig6274.3  6882-6709 | 67% |  |
| 108-168 | Contig6274.3  5192-5010 | 75% |  |
| 168-253 | No blast results |  |  |
| 253-295 | Contig6274.3  3452-3321 | 72% | cl16911, S-adenosylmethionine-dependent methyltransferases (SAM or AdoMet-MTase) |
| 311-358 | Contig6274.3  1711-1568 | 60% |  |
| 359-389 | Contig6274.3  881-789 | 80% |  |

DNMT3 tblastN results (query sequence: NP_787046.1, 845AA): No significant results

Tet1 tblastN results (query sequence: NP_085128.2, 2136AA):

| Localizations on query sequence | Localisation on bg contig sequence | % positives | Conserved domains |
| --- | --- | --- | --- |
| 0-1488 | No blast results |  | cl03401, CXXC zinc finger domain |
| 1488-1601 | Contig18827.1  4390-3917 | 51% | pfam12851, Oxygenase domain of the 2OGFeDO superfamily |
| 1604-1639 | Contig18827.1 2928-2722 | 72% |  |
| 1638-1685 | Contig18827.1 1356-1213 | 79% |  |
| 1456-1488 | Contig21.52  41322-41224 | 81% |  |
| 1488-1557 | Contig21.52  40969-40763 | 67% | pfam12851, Oxygenase domain of the 2OGFeDO superfamily |
| 1559-1601 | Contig21.52  40623-40495 | 81% |  |
| 1604-1639 | Contig21.52  38529-38422 | 81% |  |
| 1638-1685 | Contig21.52  37121-36978 | 79% |  |
| 1700-1738 | Contig21.52  35681-35565 | 81% |  |
| 1738-1996 | No blast results |  |  |
| 1996-2062 | Contig21.52  33830-33630 | 76% |  |

Tet2 tblastN results (query sequence: NP_001120680.1, 2002AA)

| Localizations on query sequence | Localisation on bg contig sequence | % positives | Conserved domains* |
| --- | --- | --- | --- |
| 0-1250 | No blast results |  |  |
| 1199-1311 | Contig18827.1  4390-3917 | 55% | pfam12851, Oxygenase domain of the 2OGFeDO superfamily |
| 1343-1395 | Contig18827.1 1371-1213 | 75% |  |
| 1319-1349 | Contig18827.1 2814-2722 | 70% |  |
| 1096-1167 | 43136-42942 | 76% |  |
| 1167-1199 | Contig21.52  41322-41224 | 84% |  |
| 1199-1268 | Contig21.52  40969-40760 | 71% |  |
| 1269-1311 | Contig21.52  40623-40495 | 86% | pfam12851, Oxygenase domain of the 2OGFeDO superfamily |
| 1319-1349 | Contig21.52  38514-38422 | 70% |  |
| 1343-1395 | Contig21.52  37136-36978 | 75% |  |
| 1409-1446 | Contig21.52  35684-35511 | 68% |  |
| 1849-1914 | Contig21.52  33830-33633 | 78% | pfam12851, Oxygenase domain of the 2OGFeDO superfamily |

Tet3 tblastN results (query sequence: NP_659430.1, 1660AA)

| Localizations on query sequence | Localisation on bg contig sequence | % positives | Conserved domains* |
| --- | --- | --- | --- |
| 1-759 | No blast results |  |  |
| 759-886 | Contig18827.1  4390-3887 | 52% | pfam12851, Oxygenase domain of the 2OGFeDO superfamily |
| 1343-1395 | Contig18827.1 1371-1213 | 75% |  |
| 1319-1349 | Contig18827.1 2814-2722 | 70% |  |
| 1096-1167 | 43136-42942 | 76% |  |
| 1167-1199 | Contig21.52  41322-41224 | 84% |  |
| 1199-1268 | Contig21.52  40969-40760 | 71% |  |
| 1269-1311 | Contig21.52  40623-40495 | 86% | pfam12851, Oxygenase domain of the 2OGFeDO superfamily |
| 1319-1349 | Contig21.52  38514-38422 | 70% |  |
| 1343-1395 | Contig21.52  37136-36978 | 75% |  |
| 1409-1446 | Contig21.52  35684-35511 | 68% |  |
| 1849-1914 | Contig21.52  33830-33633 | 78% | pfam12851, Oxygenase domain of the 2OGFeDO superfamily |

*Conserved domains were detected by doing a specialized blast (<http://www.ncbi.nlm.nih.gov/Structure/cdd/wrpsb.cgi>) using as query protein the homo sapiens characterized proteins.

MBD1 tblastN results (query sequence: NP_001191066.1): no significant results

# MBD2 tblastN results (query sequence: AAC68871.1 411AA):

| Localizations on query sequence | Localisation on bg contig sequence | % positives | Conserved domains* |
| --- | --- | --- | --- |
| 0-154 |  |  |  |
| 154-181 | Contig7228  9946-9863 | 100% | cd01396, MeCP2, MBD1, MBD2, MBD3, and MBD4 are members of a protein family that share the methyl-CpG-binding domain (MBD) |
| 179-224 | Contig7228  7409-7272 | 78% |  |
| 204-305 | Contig7228  7155-6856 | 53% |  |
| 281-310 | Contig7228  6226-6137 | 83% | pfam14048, C-terminal domain of methyl-CpG binding protein 2 and 3 |
| 311-357 | Contig7228  5753-5607 | 65% |  |
| 357-411 |  |  |  |

MBD3 tblastN results (query sequence: AAC68876.1, 291AA):

| Localizations on query sequence | Localisation on bg contig sequence | % positives | Conserved domains* |
| --- | --- | --- | --- |
| 10-37 | Contig7228  9946-9863 | 85% | cd01396, MeCP2, MBD1, MBD2, MBD3, and MBD4 are members of a protein family that share the methyl-CpG-binding domain (MBD) |
| 35-92 | Contig7228  7409-7239 | 67% |  |
| 94-137 | Contig7228  7050-6916 | 71% | pfam14048, C-terminal domain of methyl-CpG binding protein 2 and 3 |
| 137-166 | Contig7228  6226-6137 | 80% |  |
| 167-213 | Contig7228  5753-5607 | 63% |  |

MBD4 tblastN results (query sequence: AAC68879.1, 580AA): only the carboxy terminal part of the query protein matches with a target in the *B. glabrata* genome. This carboxy terminal part of AAC68879.1 contains a domain involved in endonuclease activity (cl14786). The coverage is too low to conclude for the presence of a MBD4 in *B. glabrata* genome.
