# Supplementary figures and images for: 5-methyl-cytosine and 5-hydroxy-methyl-cytosine in the genome of Biomphalaria glabrata, a snail intermediate host of Schistosoma mansoni
Source: Parasit Vectors. 2013 Jun 6;6:167. doi: 10.1186/1756-3305-6-167 (PMC3681652; doi:10.1186/1756-3305-6-167)

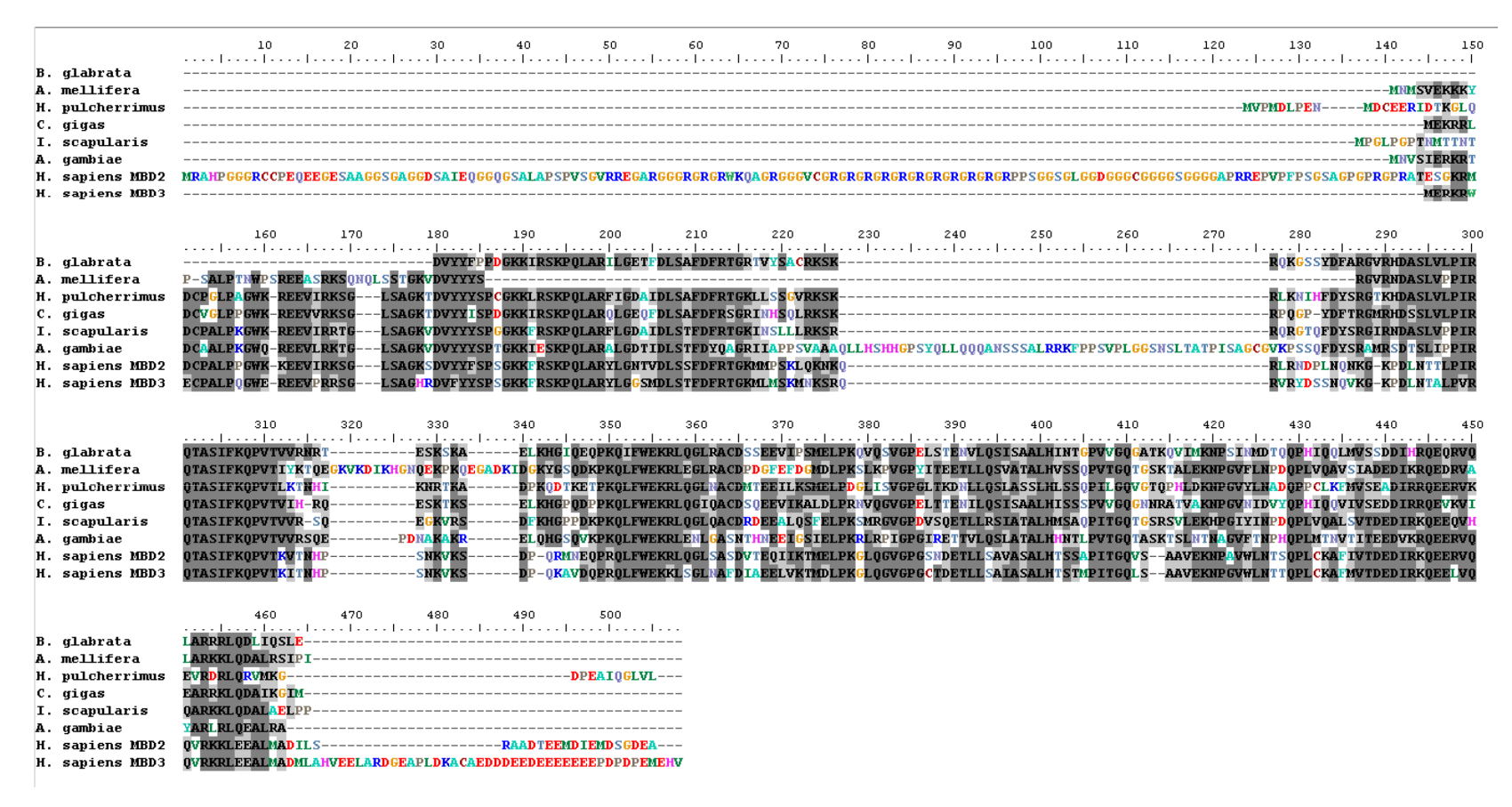

Supplement: Additional file 2 — Sequence alignments of MBD2/3 protein families.B. glabrata MBD2/3 protein sequence was aligned with the BioEdit software (http://www.mbio.ncsu.edu/bioedit/bioedit.html) with the following protein sequences: Apis mellifera MBD2/3 (XP_392422.2), Homo sapiens MBD2 (NP_003918.1), H. sapiens MBD3 (NP_003917.1), Hemicentrotus pulcherrimus MBD2/3 (ACF05485.1), Crassostrea gigas MBD2/3 (EKC32831.1), Ixodes scapularis MBD2/3 (XP_002407962.1). [file 1756-3305-6-167-S2.tiff]
